# The Hyperdense middle cerebral artery sign is associated with poor leptomeningeal collaterals in acute ischemic stroke: a retrospective study

**DOI:** 10.1186/s12883-022-02566-9

**Published:** 2022-02-11

**Authors:** Ye Hong, Jinghuan Fang, Mengmeng Ma, Wei Su, Muke Zhou, Li Tang, Huairong Tang, Li He

**Affiliations:** 1 Department of Neurology, West China Hospital of Sichuan University, Wainan Guoxue Xiang #37, Chengdu, 610041 Sichuan China; 2 Institute of Brain Science and Brain-Inspired Technology of West China Hospital, Sichuan University, Chengdu, China; 3 Department of Health Management Center, West China Hospital of Sichuan University, Wainan Guoxue Xiang #37, Chengdu, 610041 Sichuan China

**Keywords:** Acute ischemic stroke, Hyperdense middle cerebral artery sign, Leptomeningeal collaterals

## Abstract

**Background:**

The hyperdense middle cerebral artery sign (HMCAS) is an early radiological marker to provide an early diagnosis and to identify ischemia. As reported, HMCAS is associated with heavy clot burden. Moreover, a heavy clot burden may cause obstruction of the orifices of arteries for leptomeningeal collateral flows and can lead to severe clinical conditions. However, the direct relationship between HMCAS and collateral flows remains unclear. Therefore, we explored the association between HMCAS and leptomeningeal collaterals in patients with acute ischemic stroke.

**Methods:**

Consecutive ischemic stroke patients were enrolled from January 2015 to April 2021. HMCAS appearance and collateral status were detected by multimodal computed tomography at admission. Logistic regression analyses helped to identify the association between HMCAS, collateral flows and stroke severity.

**Results:**

In 494 included patients, 180 (36.4%) presented with HMCAS. Ipsilateral collaterals were not seen or less prominent in patients with HMCAS (*P* < 0.001). The HMCAS appearance was significantly associated with less collaterals (odds ratio 5.17, 95% confidence interval 3.27-8.18, *P* < 0.001), internal carotid artery + M1/M1 occlusion, the initial stroke severity and follow-up outcomes. Subgroup analyses further confirmed HMCAS as an indicator of poor collaterals in ischemic stroke (all *P* values < 0.05).

**Conclusions:**

HMCAS is associated with poor leptomeningeal collaterals, the stroke severity and a poor neurological outcome. Therefore, the HMCAS appearance can act as an early warning sign for healthcare professionals to be alert for poor collateral flows and poor neurological outcomes in ischemic stroke patients with middle cerebral artery occlusion.

## Background

Acute ischemic stroke is the second leading cause of death worldwide [1]. Patients often suffer from neurologic disability and severe psychological distress, and these patients have an increased risk of rehospitalization due to complications. Leptomeningeal collateral flows are pre-existing anastomoses that are capable of maintaining the blood flow to the infarct region [2]. After cerebral ischemia, collaterals provide supplementary blood flow to save the brain tissue from irreversible damage [3]. Patients with poor leptomeningeal collateral flows usually have a higher severity of stroke and worse clinical outcomes in acute ischemic stroke [4, 5]. The condition of collateral blood vessels has become a key target for stroke treatment.

In some patients with a large cerebral artery occlusion, early noncontrast computed tomographic (NCCT) scans could visualize a region of hyperdensity in comparison to the artery on the contralateral side. Although the hyperdense artery sign has been reported in the internal carotid artery and posterior cerebral artery basilar artery, it is most widely described in the middle cerebral artery, termed the hyperdense middle cerebral artery sign (HMCAS) [6, 7]. HMCAS is a radiological marker of intra-arterial thromboembolism and can provide early diagnosis to identify ischemia before the infarct becomes visible and before brain damage develops [8–11].

To date, several studies have shown that HMCAS is the result of special clot features. The thrombus volumes are larger and longer in patients who have the hyperdense sign than in those without, suggesting that HMCAS is associated with a heavy clot burden in ischemic stroke [12, 13]. Since a heavy clot burden can easily cause obstruction of the orifices of arteries for leptomeningeal collateral flows, HMCAS may be closely related to the collaterals and angioarchitecture in acute ischemic stroke [13]. However, there is no direct relationship between the appearance of the hyperdense artery sign and collateral flows.

Thus, we conducted this study to demonstrate the association between the presence of HMCAS and leptomeningeal collateral circulation in ischemic stroke patients with unilateral middle artery occlusion. The knowledge thus gained could provide more evidence for cerebral blood flow during the acute phase after stroke onset.

## Methods

### Participants

This retrospective study was conducted at West China Hospital, SCU, from January 2015 to April 2021. The inclusion criteria were a. patients were ≥ 18 years of age, b. patients who had acute ischemic stroke diagnosed on admission according to the World Health Organization (WHO) criteria [14], c. patients who had NCCT and CT angiography (CTA) images within 12 h of symptom onset that were available, and d. patients who had CTA detection of acute infarction due to a unilateral occlusion of the MCA (with or without occlusion of the internal carotid artery). The exclusion criteria included a prestroke modified Rankin Scale (mRS) score > 1, b. atherosclerotic extracranial ipsilateral carotid artery stenosis≥50% detected by CTA, c. already taken thrombolysis or endovascular treatment elsewhere before administration.

### Data collection

#### General information and clinical data

Clinical variables, including sex and age, stroke risk factors, premorbid medications (hypotensive, hypoglycemic, lipid-lowering, anti-platelet, and anticoagulation therapy), and biochemical indexes, were collected from the hospital stroke database. The severity of neurologic deficits at admission and the functional outcomes at 90 days after discharge were assessed by the National Institutes of Health Stroke Scale (NIHSS) score and the mRS score, respectively.

#### Evaluation of HMCAS and baseline collaterals

All patients underwent both noncontrast CT and CTA of the head on admission at the radiology center of West China Hospital, SCU. The CT examinations were performed by 128-row dual-source CT (Somatom Definition FLASH, Siemens Healthcare, Germany) with following parameters: 70 kV, 150 mA, and 5-mm collimation for CTs; 6 ml/s contrast with 42 ml in total, a 3 to 5 s delay from injection to scanning, a 1.25-mm thick slice, and the same voltage and electricity standard were used as were used for the previous CTAs. The original images were transferred to the workstation, and 3D images of head and neck blood vessels were reconstructed by software. By combining multiplanar reconstruction, maximum intensity projection and other postprocessing techniques, the occlusion of large vessels in anterior circulation could be observed.

HMCAS was defined as a unilateral appearance of higher density of the MCA than that of the surrounding brain on the unenhanced CT scan [10]. In addition, HMCAS should be considered when patients fulfilled these criteria: (1) spontaneous visibility of the horizontal part of the MCA, (2) disappearance of HMCAS after adjustment of the scans to bone window levels and (3) absence of subarachnoid bleeding he increased density on the bone window density should disappear [10]. Two radiologists reviewed all of the NCCT images to identify HMCASs. Another two physicians who were blinded to the results of both HMCAS identification and clinical information assessed the collateral flows in the CTA images based on the regional leptomeningeal collateral scores (rLMCs). An rLMC score of 0-10 was defined as poor collateral flows, 11-16 as moderate and 17-20 as good [4]. A third evaluator with extensive experience would then make a final assessment to deal with any disagreements. Based on the presence of HMCAS, the patients were divided into an HMCAS group and a non-HMCAS group.

### Follow-up and outcomes

The patients were followed up for 90 days after discharge. The primary outcome was the association between the collateral flows and HMCAS. The secondary outcomes were the association between HMCAS, the stroke severity and the clinical outcomes.

### Subgroup analyses

Subgroup analyses were further conducted after the patients were stratified by age, sex, thrombus location and stroke type, and the subgroups analyses were used to verify the potential association between existential HMCAS and collateral flows.

### Statistical analyses

Comparisons were performed using SPSS, Version 23.0 (IBM, Chicago, IL, USA). Normally distributed variables were reported as the mean ± standard deviation and calculated by Student’s *t* tests; nonnormally distributed variables were analyzed using Pearson’s chi-square test or Mann–Whitney U tests and were reported as the median (IQR). Categorical variables are presented as frequencies (percentages) and were analyzed using the chi-square test or Fisher’s exact test. Univariate logistic regression helped to identify potential determinants of poor collateral flows, and a multivariate logistic regression was used to determine the association between collaterals and the presence of HMCAS. All significant univariate variables were adjusted step by step from model 1 to model 4. Model 1 was adjusted for age and sex; model 2 included model 1 and was additionally adjusted for the stroke risk factors; model 3 included model 2 plus the thrombus location on CTA; and model 4 included model 3 and the admission examinations (all significant univariate variables). Logistic regression was used to determine the clinical outcomes associated with HMCAS. Subgroup analyses were further conducted to verify the association between HMCAS and poor collateral flows. All *P* values are two-sided, with a *P* value < 0.05 considered statistically significant.

## Results

### Demographic and clinical characteristics

There were 599 patients with documented anterior circulation occlusions on the baseline CTAs. After excluding 49 patients for simultaneously detected occlusion in the contralateral MCA or posterior circulation, 14 patients for having over 10% missing data, 11 patients for poor image data and 31 patients for taking reperfusion therapies elsewhere before administration, 494 patients were included for the analyses. Two hundred and seventy-eight (56.3%) patients were male. HMCAS was identified in 180 (36.4%) patients. Compared to the non-HMCAS group, the HMCAS group tended to have a history of atrial fibrillation (AF) (*P* < 0.001), a higher stroke severity (*P* < 0.001), a lower systolic blood pressure (*P* = 0.004), a proximal occlusion (internal carotid artery + M1/M1 occlusion) (*P* < 0.001), a higher proportion of endovascular treatment (*P* < 0.001) and poor collaterals (*P* < 0.001). Good collaterals (*P* < 0.001) were less likely to be seen in HMCAS patients (Table 1).

**Table 1:** Baseline characteristic of stroke patients with/without HMCAS

Variables	Present with HMCAS		
	Yes(*n* = 180)	No(*n* = 314)	*P* value
Characteristics			
Age (in yrs., mean ± SD)	67.9 ± 13.6	66.4 ± 13.8	0.262
Male (n, %)	91 (50.6)	187 (59.6)	0.052
Risk Factors, n (%)			
Hypertension	90 (50.0)	177 (56.4)	0.172
Diabetes mellitus	41 (22.8)	72 (22.9)	0.969
Atrial fibrillation^a^	103 (57.2)	100 (31.8)	< 0.001
Coronary heart disease	20 (11.1)	23 (7.3)	0.151
Previous stroke/TIA	17 (9.4)	49 (15.6)	0.053
Dyslipidemia	19 (10.6)	45 (14.3)	0.229
Current smoking	28 (15.6)	45 (14.3)	0.712
Premorbid Drug Use, n (%)			
Antithrombotic	21 (11.7)	22 (7.0)	0.077
Antiplatelet	11 (6.1)	25 (8.0)	0.446
Statin	11 (6.1)	17 (5.4)	0.747
Antihypertensive	61 (33.9)	114 (36.3)	0.589
Antidiabetics	15 (8.3)	34 (10.8)	0.372
Laboratory Studies, mean ± SD			
Systolic blood pressure^a^, mmHg	140.2 ± 23.3	147.0 ± 25.8	0.004
Temperature, ^o^C	36.5 ± 0.5	36.4 ± 0.3	0.058
Blood glucose, mmol/L	8.0 ± 2.7	8.1 ± 3.1	0.675
Triglycerides, mmol/L	1.5 ± 1.0	1.7 ± 1.2	0.111
Total cholesterol, mmol/L	4.3 ± 1.1	4.3 ± 1.1	0.684
HDL-C, mmol/L	1.3 ± 0.4	1.3 ± 0.4	0.661
LDL-C, mmol/L	2.6 ± 1.0	2.5 ± 0.8	0.539
Serum uric acid, μmol/L	350.3 ± 101.1	354.8 ± 106.0	0.640
White cell count (^*^10^9/L)	8.8 ± 3.0	8.5 ± 8.9	0.639
Serum creatinine, μmol/L	74.0 ± 23.2	78.7 ± 35.3	0.112
NIHSS score at admission^a^ (median, IQR)	16 (11-20)	7 (3-15)	< 0.001
Proximal occlusion^a^	135 (75.0)	159 (50.6)	< 0.001
Collateral Status, n (%)			
rLMC 0-10^a^	121 (67.2)	72 (22.9)	< 0.001
rLMC 11-16	37 (20.6)	64 (20.4)	0.963
rLMC 17-20^a^	22 (12.2)	178 (56.7)	< 0.001
Reperfusion therapy, n (%)			
Thrombolysis	41 (22.8)	78 (24.0)	0.606
Onset-to-treatment time (r-tPA), min	191.1 ± 43.3	182.2 ± 48.1	0.338
Endovascular treatment	51 (28.3)	31 (9.9)	< 0.001
Onset-to-treatment time, min	269.9 ± 117.0	215.3 ± 73.2	0.062

*P* is calculated by t test, Chi-square test, or Mann-Whitney U test as appropriate

^a^ Variables showed significant differences

*SD* Standard Deviation, *IQR* Interquartile Range, *HMCAS* Hyperdense middle cerebral artery sign, *TIA* Transient Ischemic Attacks, *HDL-C* High density lipoprotein cholesterol, *LDL-C* Low density lipoprotein cholesterol, *NIHSS* National Institutes of Health Stroke Scale*, rLMC* regional Leptomeningeal Collateral scores*, r-tPA* recombinant tissue plasminogen activator

### Factors associated with a poor collateral grade

Table 2 shows the association between the clinical variables and the collateral status as assessed by the rLMC scores. The collaterals were categorized either as poor, moderate or good. Older age (*P <* 0.001), female sex (*P* = 0.007), previous AF (*P* < 0.001) and coronary heart disease (CHD) (*P* = 0.045), proximal occlusion (*P* < 0.001), elevated blood glucose (*P* = 0.030) and HMCAS presence (*P* < 0.001) might reduce the collateral flows. In the multivariate logistic regression, the association between HMCAS and poor collaterals was statistically confirmed from models 1 through 4. After adjusting for the significant univariate variables including age, sex, stroke risk factors (histories of atrial fibrillation and coronary heart disease), proximal occlusion and admission examinations (blood glucose and triglycerides), model 4 revealed that the presence of HMCAS (odds ratio [OR] 5.17, 95% confidence interval [CI] 3.27-8.18, *P* < 0.001) was significantly associated with less collateral flow (Table 3).

**Table 2:** Associated factors with collateral flows in acute stroke patients

Variables	Poor	Moderate	Good	*P* value	Univariate logistic analysis
	(*n* = 193)	(*n* = 101)	(*n* = 200)		Poor vs. not poor rLMC
Age > 60y	145 (75.1)	62 (61.4)	125 (62.5)	0.011	2.18 (1.43,3.33)^a^
Sex(female)	99 (51.3)	51 (50.5)	66 (33.0)	< 0.001	1.66 (1.15,2.39)^a^
Hypertension	111 (57.5)	46 (45.5)	110 (55.0)	0.139	1.26 (0.87,1.81)
Diabetes mellitus	48 (24.9)	12 (11.9)	53 (26.5)	0.012	1.20 (0.79,1.81)
Dyslipidemia	23 (11.9)	7 (6.9)	34 (17.0)	0.042	1.17 (0.68,2.01)
Atrial fibrillation	115 (59.6)	50 (49.5)	38 (19.0)	< 0.001	3.57 (2.44,5.22)^a^
CHD	23 (11.9)	6 (5.9)	14 (7.0)	0.122	1.90 (1.01,3.57)^a^
Current smoking	30 (15.5)	16 (15.8)	27 (13.5)	0.803	1.10 (0.67,1.83)
Previous stroke/TIA	20 (10.4)	15 (14.9)	31 (15.5)	0.289	0.64 (0.37,1.12)
Statins	11 (5.7)	6 (5.9)	11 (5.5)	0.988	1.01 (0.46,2.21)
SBP	150.2 ± 28.0	145.7 ± 26.1	149.2 ± 23.0	< 0.001	1.00 (0.99,1.01)
Blood glucose	8.5 ± 2.8	7.4 ± 2.3	8.0 ± 3.3	0.030	1.07 (1.01,1.14)^a^
Triglycerides	1.5 ± 0.9	1.4 ± 0.8	1.9 ± 1.4	0.033	0.82 (0.69,0.98)^a^
Total cholesterol	4.3 ± 1.0	4.2 ± 1.1	4.4 ± 1.2	0.580	0.95 (0.81,1.13)
HDL-C	1.4 ± 0.4	1.3 ± 0.4	1.3 ± 0.4	0.067	1.49 (0.97,2.29)
LDL-C	2.5 ± 0.9	2.5 ± 0.9	2.6 ± 0.9	0.510	0.93 (0.76,1.15)
Proximal occlusion	160 (82.9)	53 (52.5)	81 (40.5)	< 0.001	6.27 (4.03,9.76)^a^
HMCAS	121 (62.7)	37 (36.6)	22 (11.0)	< 0.001	6.89 (4.59,10.36)^a^

^a^showed significant differences in univariate analysis

*HMCAS* Hyperdense middle cerebral artery sign, *TIA* Transient Ischemic Attacks, *CHD* Coronary heart disease*, SBP* Systolic blood pressure, *HDL-C* High density lipoprotein cholesterol, *LDL-C* Low density lipoprotein cholesterol, *rLMC* regional Leptomeningeal Collateral scores

**Table 3:** Association between the presence of HMCAS and collaterals

	Poor rLMC vs. Not poor rLMC	
	OR (95% CI)	*P* value
Unadjusted	6.89 (4.59,10.36)	< 0.001
Model 1	7.02 (4.62, 10.66)	< 0.001
Model 2	6.13 (4.00, 9.39)	< 0.001
Model 3	4.98 (3.17, 7.84)	< 0.001
Model 4	5.17 (3.27, 8.18)	< 0.001

Model 1: Adjusted for age and gender;

Model 2: Adjusted model 1 plus stroke risk factors;

Model 3: Adjusted model 2 plus thrombus location;

Model 4: Adjusted model 3 plus admission examinations

### HMCAS is associated with the stroke severity and clinical outcomes

To test the theory that the hyperdense artery sign is an indicator of a poor neurological prognosis, our study also explored the association between the presence of HMCAS and the stroke severity and clinical outcomes. In Table 4, the data exhibited significant differences between the presence of HMCAS and the stroke severity and the follow-up mRS. Patients with HMCAS were less likely to have a minor or a moderate stroke (NIHSS score 0-5 vs. ≥ 5, OR 0.15, 95% CI 0.08-0.29, *P* < 0.001; NIHSS score 0-15 vs. ≥ 15, OR 0.27, 95% CI 0.18-0.41, *P* < 0.001). Moreover, the appearance of the hyperdense artery sign in the acute phase of stroke was significantly associated with a worse prognosis at 90 days. Even taking timely and successful reperfusion therapies (thrombolysis or endovascular treatment), patients with HMCAS generally failed to have good outcomes (mRS scores 0-3, OR 0.35 95% CI 0.21-0.58, *P* < 0.001 for intravenous thrombolysis and OR 0.34 95%CI 0.20-0.56, *P* < 0.001 for endovascular treatment) or favorable outcomes (mRS scores 0-2, OR 0.23 95% CI 0.13-0.40*, P* < 0.001 for intravenous thrombolysis and OR 0.21 95%CI 0.12-0.37, *P* < 0.001 for endovascular treatment). The thrombus location was also statistically significant among the patients in the two groups. The appearance of HMCAS indicated proximal occlusion in stroke patients (OR 1.72 95%CI 1.10-2.71, *P* = 0.018).

**Table 4:** Association between HMCAS present and stroke severity and clinical outcomes

	Univariate analysis		Multivariate analysis	
	OR (95% CI)	*P* value	OR (95% CI)	*P* value
NIHSS scores				
0-5 vs. ≥ 5	0.13 (0.07, 0.24)	< 0.001	0.15 (0.08, 0.29)	< 0.001
0-15 vs. ≥ 15	0.22 (0.15, 0.33)	< 0.001	0.27 (0.18, 0.41)	< 0.001
Thrombus location				
ICA + M1/M1 vs. M2	3.01 (2.01, 4.52)	< 0.001	1.72 (1.10,2.71)	0.018
90d mRS scores				
0-3 vs. 4-6	0.17 (0.11, 0.25)	< 0.001	0.35 (0.21, 0.58) ^a^	< 0.001
			0.34 (0.20,0.56) ^b^	< 0.001
0-2 vs. 3-6	0.11 (0.07, 0.18)	< 0.001	0.23 (0.13, 0.40) ^a^	< 0.001
			0.21 (0.12,0.37) ^b^	< 0.001

For NIHSS scores: Adjusted for age, gender, thrombus location in multivariate analysis

For Thrombus location: Adjusted for age, gender, NIHSS score in multivariate analysis

For 90d mRS score: ^a^ Adjusted for age, gender, thrombus location, NIHSS score, thrombolysis treatment in multivariate analysis; ^b^ Adjusted for age, gender, thrombus location, NIHSS score, endovascular treatment in multivariate analysis

*NIHSS* National Institutes of Health Stroke Scale, *ICA* internal carotid artery, *mRS* modified Rankin Scale

Moreover, in Table 5, we also explored the association between HMCAS persistent on follow-up CT scan in 22-36 h and poor clinical outcomes. HMCAS observed on the admission CT scan in 180 patients still present on the follow-up CT scan in 72 patients. Poor functional outcomes were significantly associated with persistence of HMCAS (mRS 4-6 OR 5.75, 95%CI 2.84-11.63, *P* < 0.001 and mRS 3-6 OR 4.92, 95%CI 1.98-12.18, *P* = 0.001).

**Table 5:** Association between persistence of HMCAS and poor outcome at 3 months

Variable	OR (95%CI)	*P* value
^a^Persistence of HMCAS (*n* = 72)		
mRS score 4–6 points	5.75 (2.84-11.63)	< 0.001
mRS score 3-6 points	4.92 (1.98,12.18)	0.001

^a^HMCAS was present on follow-up CT scan at 22–36 h

Adjusted for age, gender, thrombus location and NIHSS score

*mRS* modified Rankin Scale

### Subgroup analyses

**Fig. 1:**
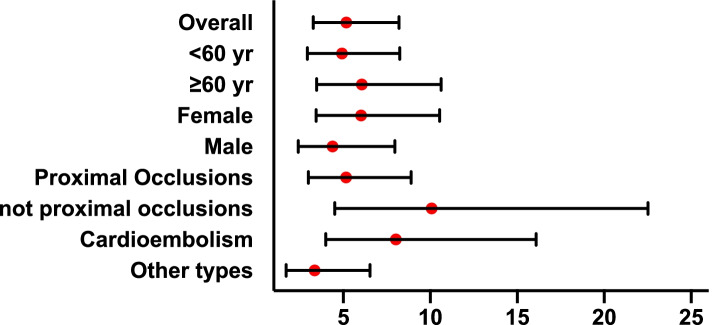
The subgroup analyses stratified by age, sex, thrombus location and stroke type

To further investigate the association between the detection of HMCAS and poor collaterals in acute ischemic stroke, we conducted subgroup analyses. Figure 1 shows the subgroup analyses after stratifying the patients by age, sex, thrombus location and stroke type. After adjustment for important confounding factors, our results showed that the detection of HMCAS was closely associated with poor leptomeningeal collateral flows in ischemic stroke patients, and this association was independent of sex, age, stroke type and thrombus location (all *P* values < 0.05).

## Discussion

We performed this retrospective study in stroke patients to explore the association between the presence of HMCAS and poor collaterals before medical intervention. Our findings showed that the presence of HMCAS was associated with poor leptomeningeal collaterals among acute ischemic stroke patients who had a stroke that was caused by major arterial occlusion.

The present study provided new evidence between HMCAS and leptomeningeal collaterals. HMCAS is an indicator of the presence of an arterial thrombus in acute ischemic stroke. Classification of thrombi as red blood cell dominant was noted in every case in which HMCAS was identified [15]. The “red clots” with rich erythrocytes and higher hemoglobin concentrations can present with increased attention on the CT images [15]. As reported, the thrombus volume is significantly larger in patients with HMCAS than in those without HMCAS in the proximal occlusion [12]. Larger, longer clots with a heavier clot burden will interfere with collateral flows in bypass vessels and can cause severe tissue injury [12, 15]. A study found that the absence of HMCAS predicted better leptomeningeal collaterals in the univariate analysis. It also mentioned that better collateralization was independently associated with a lower clot burden [16]. In accordance with these previous findings, we found that the presence of HMCAS can provide additional information by indicating the obstruction of collateralization when these patients are compared with patients without HMCAS. This may explain why HMCAS is a negative sign for the stroke severity and clinical outcomes. Nevertheless, additional studies in a larger population are necessary to corroborate our observations.

In this study, patients with HMCAS showed a tendency to have a proximal occlusion. According to the literature, a proximal MCA occlusion in the anterior circulation could result in worse collateral flow than a distal occlusion [5, 16]. A proximal MCA occlusion can cause larger ischemic areas and less adequately perfused brain tissue surrounding it, hence leading to sufficient collaterals [17]. In addition, when CTA is performed, the time for contrast arrival via leptomeningeal collaterals might be longer for patients with a proximal occlusion, which could result in less collateralization in the CTA images. Similarly, patients with HMCAS showed a high proportion of premorbid AF [18]. Patients with cardioembolic occlusions are thought to be associated with a paucity of previously developed collaterals leading to more severe hypoperfusion and larger infarction areas [19, 20]. A neuroimaging study showed that patients with acute cardiogenic cerebral embolism had a larger volume of severe hypoperfusion at baseline, suggesting less leptomeningeal collateral flow [20]. Therefore, this evidence may explain the reason why patients with HMCAS usually have less collateral flow.

Leptomeningeal arterial collaterals can provide alternative blood flow that can support the brain viability during an acute ischemic stroke. A greater degree of baseline collaterals is associated with a smaller infarct size and improved recanalization after endovascular treatment [21, 22]. In contrast, poor leptomeningeal collateral flow is related to severe conditions after a stroke occurs. An understanding of conditions associated with the development of cerebral collaterals is increasingly important in ischemic stroke treatment. Previous studies have found that genetic factors, prior hypertension, age, premorbid drug use, metabolic syndrome and circle of Willis completeness may affect the patency of leptomeningeal collaterals [23–27]. In the present study, the presence of HMCAS showed a significant association with fewer collateral flows, and this was independent of age, sex, stroke risk factors, initial physical examination and thrombus location (model 4). Moreover, Consistent with former studies, HMCAS can also indicate that there is a proximal occlusion, a higher stroke severity and worse clinical outcomes in the multivariable analysis [5–11, 28]. Patients with persistent HMCAS on the follow-up CT are supposed to have poor functional outcomes [29]. Our findings have pointed out that HMCAS can act as an early warning sign for poor collateralization and a worse prognosis in ischemic stroke patients.

Our study also has several limitations. First, the present study is a nonrandomized observational single-center study that recruited acute ischemic stroke patients in a tertiary hospital in East Asia, and only a few patients underwent reperfusion therapies. However, our data are consistent with a growing body of evidence suggesting that the appearance of HMCAS can predict a poor clinical outcome and can support the hypothesis that the hyperdense artery sign in MCA can indicate less leptomeningeal collateral flow in patients with ischemic stroke. Nevertheless, studies that evaluate more ethnic groups, effect of reperfusion therapies and multicenter studies are needed in the future. Second, the thrombus size was not recorded, which may have caused different effects on the collaterals and clinical outcomes. However, as a result of the generally heavy clot burden in HMCAS, this would not essentially change our results. As such, more research on the detailed association between the hyperdense artery sign and leptomeningeal collaterals is needed in the future. Further preclinical studies on the mechanisms of HMCAS appearance in arteriogenesis are also needed. Third, collaterals have been only evaluated by rLMC scores on the admission CTA scans. It should be noted that the predictive value for clinical outcomes varies among different score systems, and the temporal evolution of collateral flows plays an important role in the determining the outcomes after stroke onset [30, 31]. Since the rLMC score has been verified as a strong imaging parameter on admission CTA for predicting clinical outcomes in stroke patients, our results would not be essentially changed [32]. Future studies may evaluate collaterals in various ways for accuracy and focus on the temporal evolution of intracranial collaterals on the follow-up CTA. Fourth, the ASPECTS of the patients and the topography of the collateral status were not recorded. The effect of large early ischemic damage determined by ASPECTS can be overcome by good collaterals and collateral status of specific region may serve as a more reliable biomarker for prognosis as reported [33, 34]. Nevertheless, the rLMC score we used is based on the major anatomic regions of the anterior circulation that is comparable to the ASPECTS system, so the findings still possess a certain reference value [35]. Future studies should pay more attention on the early ASPECTS and topological collateral scores when evaluating the collateral flows and clinical outcomes.

## Conclusion

Our study demonstrated the direct relationship between the appearance of the hyperdense artery sign and collateral flows. The present study showed that HMCAS is associated with poor leptomeningeal collaterals, the stroke severity and poor neurological outcomes during acute stroke. Therefore, HMCAS can act as an early warning sign for healthcare professionals to be alert for poor collateral flows and poor neurological outcomes in ischemic stroke patients with a middle cerebral artery occlusion.

## Data Availability

The data that support the findings of this study are available from the corresponding author upon reasonable request.

## Abbreviations

HMCAS: Hyperdense Middle Cerebral Artery Sign
MCA: Middle cerebral artery
WHO: World Health Organization
mRS: Modified Rankin Scale
NIHSS: National Institutes of Health Stroke Scale
NCCT: Non-contrast computed tomographic
CTA: CT angiography
rLMC: Regional leptomeningeal collateral scores
AF: Atrial fibrillation
CHD: Coronary heart disease
OR: Odds ratio
CI: Confidence interval
